# Molecular phylogeny of Myriapoda provides insights into evolutionary patterns of the mode in post-embryonic development

**DOI:** 10.1038/srep04127

**Published:** 2014-02-18

**Authors:** Hideyuki Miyazawa, Chiaki Ueda, Kensuke Yahata, Zhi-Hui Su

**Affiliations:** 1 Department of Biological Sciences, Graduate School of Science, Osaka University, Osaka 560-0043, Japan; 2 JT Biohistory Research Hall, 1-1 Murasaki-cho, Takatsuki, Osaka 569-1125, Japan; 3 Graduate School of Life and Environmental Sciences, University of Tsukuba, Tsukuba, Ibaraki 305-8572, Japan

## Abstract

Myriapoda, a subphylum of Arthropoda, comprises four classes, Chilopoda, Diplopoda, Pauropoda, and Symphyla. While recent molecular evidence has shown that Myriapoda is monophyletic, the internal phylogeny, which is pivotal for understanding the evolutionary history of myriapods, remains unresolved. Here we report the results of phylogenetic analyses and estimations of divergence time and ancestral state of myriapods. Phylogenetic analyses were performed based on three nuclear protein-coding genes determined from 19 myriapods representing the four classes (17 orders) and 11 outgroup species. The results revealed that Symphyla whose phylogenetic position has long been debated is the sister lineage to all other myriapods, and that the interordinal relationships within classes were consistent with traditional classifications. Ancestral state estimation based on the tree topology suggests that myriapods evolved from an ancestral state that was characterized by a hemianamorphic mode of post-embryonic development and had a relatively low number of body segments and legs.

The subphylum Myriapoda is a terrestrial arthropod group comprising four classes: Chilopoda (centipedes), Diplopoda (millipedes), Pauropoda (pauropods), and Symphyla (symphylids) (Fig. 1). Of these, the Chilopoda, which is represented by 5 extant orders and approximately 5,000 known species^1^, is the only predatory myriapod group. A key trait of this group is a pair of poison claws formed from a modified first appendage. The Diplopoda is the most diverse myriapod group, consisting of 16 extant orders and more than 12,000 known species^1^. This group is characterized by the diplosegments in which two pairs of legs are arranged on one body segment. The remaining two classes, Pauropoda and Symphyla, are small, translucent, soil-dwelling myriapods, with body lengths of less than 2 mm and 1-8 mm, respectively. The symphylids have long and filiform antennae, and a pair of specialized appendages at the preanal segment, called spinnerets, while the pauropods have distinctive antennae, which are branching and have long flagella. 835 pauropod species in 2 orders and 5 families, and 195 symphylid species in one order and 2 families have been described to date^2^,^3^.

Most myriapods acquire additional segments and legs during post-embryonic development. Four general modes of post-embryonic development are recognized in extant myriapods^4^,^5^, including epimorphosis, euanamorphosis, hemianamorphosis, and teloanamorphosis. Of these, the first 3 modes are characterized by molts that occur throughout the life of the myriapods, while the last 3 modes, known as anamorphosis, are characterized by increasing the number of body segments. In epimorphosis, no addition of new body segments occurs at the time of molting. In euanamorphosis, every molt is characterized by the addition of new segments. In hemianamorphosis, initial molts are characterized by the addition of new segments but no further segments are added once a maximum number of segments is attained. In teloanamorphosis, molt and segment addition both cease at a certain stage and no further molts or segment addition occur thereafter (Supplementary Fig. S1)^5^,^6^. While myriapods within the same order appear to have a common mode of post-embryonic development, the mode is not conserved at the class level, especially diverse in diplopods^4^,^5^. In order to understand the diversity and evolutionary patterns of the post-embryonic development mode in myriapods, it is fundamental to clarify myriapod phylogeny.

Myriapods were traditionally considered to be paraphyletic with hexapods based on morphological and developmental studies, and for a long time this was the only nearly universally accepted results in the relationships among major arthropod lineages^7^. This traditional view, however, has been rejected by molecular-based studies that show a strong affinity between crustaceans and hexapods, and these two groups together are now widely referred to as Pancrustacea^8^,^9^,^10^. A recent morphology-based study also supports the validity of Pancrustacea^11^. On the other hand, the phylogenetic position of Myriapoda within Arthropoda remains unclear. Numerous molecular phylogenetic analyses support the Mandibulata hypothesis which proposes that the Myriapoda is a sister lineage to Pancrustacea^10^. Alternatively, the Myriochelata hypothesis, which is based on both molecular and developmental evidence, suggests that Myriapoda and Chelicerata are sister lineages^8^,^12^.

Within Myriapoda, some molecular phylogenetic analyses have suggested that the myriapods are paraphyletic or polyphyletic in relation to the chelicerates^9^,^13^, however, recent studies strongly support the monophyly of myriapods^10^,^14^,^15^,^16^. Although monophyly of each class is also supported by both morphological and molecular evidence^14^,^17^, the relationships among the classes are controversial. For example, numerous morphological and developmental studies have consistently shown that Pauropoda and Diplopoda are sister lineages, and as a result, these two classes have been grouped together as Dignatha^17^,^18^,^19^,^20^. Dignatha and Symphyla have traditionally been classified into the taxon Progoneata (Fig. 2a)^17^,^19^,^20^, but an affinity between Symphyla and Chilopoda has also been suggested based on the structure of the second maxilla (Fig. 2b)^18^. In contrast to this traditional view, molecular analyses based on 18S and 28S rDNA sequences have shown that Pauropoda and Symphyla are sister clades^15^, a relationship that is supported by other molecular analyses based on nuclear and mitochondrial protein-coding genes^10^,^21^. The latter two studies also support monophyly of the Progoneata (Fig. 2c), but the relatively few samples and the possibility of a long-branch attraction (LBA) artifact in the results obtained from the rDNA sequences^15^. Regier et al. (2005) attempted to clarify the phylogeny of myriapods using three nuclear protein-coding genes and a wide variety of samples, but the relationships between the classes could not be resolved^14^.

The following interordinal relationships within the class Chilopoda have been proposed based on morphological and developmental analyses: (Scutigeromorpha, (Lithobiomorpha, (Craterostigmomorpha, (Scolopendromorpha, Geophilomorpha))))^17^,^22^,^23^,^24^,^25^. However, the results inferred from molecular analyses are either equivocal or inconsistent^10^,^23^,^24^,^25^, even though monophyly of the orders is supported by mitochondrial and nuclear rDNA sequences^26^.

The class Diplopoda is classified into two subclasses: the soft-bodied Penicillata, including only the order Polyxenida, and the Chilognatha, which have calcified exoskeletons. The latter subclass is further split into two infraclasses: the Pentazonia (three orders) and the Helminthomorpha, which is divided into the Eugnatha (seven orders) and the Colobognatha (four orders)^27^,^28^,^29^. Molecular evidence supports the monophyly of the three higher taxa (Colobognatha, Helminthomorpha, and Pentazonia), but interordinal relationships within the taxa remain unclear^14^.

The aim of this study was therefore to clarify the interclass and interordinal relationships of myriapods, and then clarify the evolutionary patterns of post-embryonic development mode in myriapods. Three nuclear protein-coding genes, the catalytic subunit of DNA polymerase delta (DPD1) and the two largest subunits of RNA polymerase II (RPB1 and RPB2) were used in phylogenetic analyses with maximum likelihood and Bayesian methods. A comprehensive samples, including 19 myriapod species encompassing 17 orders and all classes, and 11 outgroup species, including three chelicerates, three crustaceans, and five hexapods, were used in the phylogenetic analyses. An ancestral state estimation was performed based on the phylogenetic tree.

## Results

### Phylogeny of myriapods

The sequence lengths of the three genes determined in this study were: >1,900 bp for DPD1, >4,000 bp for RPB1, and >2,900 bp for RPB2. The predicted amino acid (aa) sequences were aligned together with those of the outgroup species, and the concatenated alignment for phylogenetic analysis comprised a total of 2,904 aa (611 aa for DPD1, 1319 aa for RPB1, and 974 aa for RPB2). The analyses of variable and parsimony-informative aa sites showed that DPD1 was most variable and informative, while RPB2 was the most conserved and least informative (Supplementary Table S1). These findings corroborated those obtained from sequence data for hexapods^30^,^31^.

Phylogenetic analyses with maximum likelihood (ML) and Bayesian inference (BI) generated trees with the same topologies (Fig. 3). Monophyly of the myriapods was well supported with a high bootstrap percentage (BP = 100) and posterior probability (PP = 1.00). The monophyletic origin of myriapod classes was also strongly supported, except for Diplopoda. For the interclass relationships, the resulting trees with high support values (BP = 88 and PP = 1.00) revealed that the class Symphyla is a sister lineage to the other classes (Chilopoda, Diplopoda, and Pauropoda), suggesting that Symphyla is the most basal lineage of myriapods and that the remaining three classes evolved from a common ancestor. However, the relationships among the remaining three classes could not be resolved due to low node support.

In the tree topology shown in Fig. 3, pauropods showed relatively long branches compared with other taxa. To eliminate the possibility of long-branch attraction (LBA) artifacts, we conducted phylogenetic analysis based on a dataset that excluded the sequence data of pauropods. The resulting tree showed the same topology as those obtained from the original analysis (Supplementary Fig. S2). This result suggests that the long-branch pauropods do not introduce LBA artifacts into the phylogenetic analyses.

**Figure 2:**
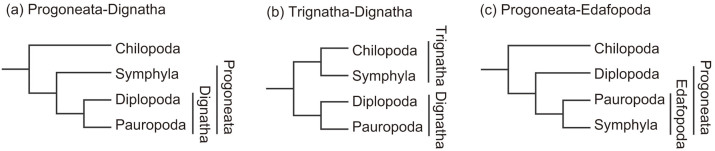
Major hypotheses for the relationships among myriapod classes. (a, b) Traditional views based on morphology^18^,^20^. (c) Hypotheses based on molecular analyses^10^,^21^,^39^.

The topology inferred for interclass relationships in this study differed from relationships proposed to date (see Fig. 2). To evaluate those previously proposed hypotheses shown in Fig. 2, a statistical analysis was performed using CONSEL^32^ with various tests, AU, KH, SH, wKH, and wSH. The results showed that *P* values obtained from the different tests were less than 0.01, except for wSH, which had a *P* value for the Trignatha-Dignatha hypothesis (Fig. 2b) of 0.018 (Table 1). Thus, using our dataset, these results indicated that the previously proposed hypotheses were rejected almost at a 1% level of significance.

**Figure 3:**
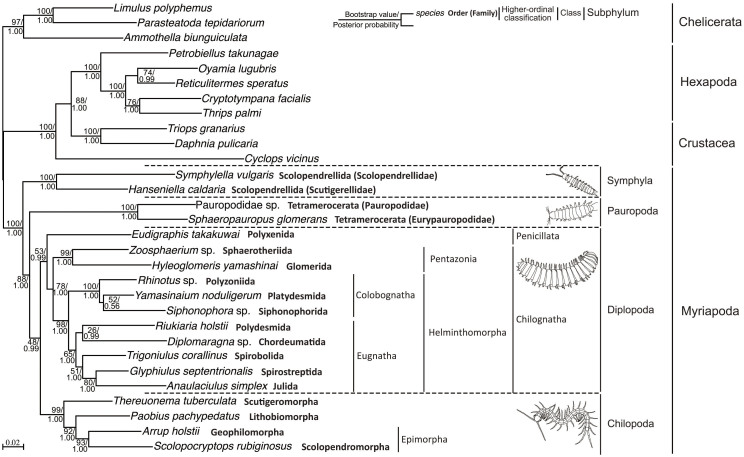
ML tree of Myriapoda based on the combined amino acid sequences of DPD1, RPB1, and RPB2. ML bootstrap values (top) and Bayesian posterior probability (bottom) are shown at each node. Bold letters after the species name indicate order name. Higher taxon names of Myriapoda are indicated to the right of the tree. The illustrations of the four representative myriapods were drawn by the first author (H.M.) based on the pictures shown in Fig. 1.

Within the class Chilopoda, our analyses strongly support the following interordinal relationships: (Scutigeromorpha, (Lithobiomorpha, (Scolopendromorpha, Geophilomorpha))). The BP and PP values for each node were >90 and 1.00, respectively (Fig. 3). The class Diplopoda consists of two subclasses, Penicillata and Chilognatha. The former includes only one order, Polyxenida, and the latter comprises all of the remaining orders. The monophyly of Chilognatha was well supported (BP = 78; PP = 1.00) (Fig. 3). Chilognatha is traditionally classified into two infraclasses, Pentazonia and Helminthomorpha, and Helminthomorpha is further classified into two groups, Colobognatha and Eugnatha. The phylogenetic trees generated in this study all supported the monophyly of these higher taxa (Fig. 3). Within Eugnatha, a sister relationship between the orders Julida and Spirostreptida was strongly supported (BP = 80; PP = 1.00), but the other interordinal relationships remain unclear due to weak support (Fig. 3).

### Divergence time among myriapod clades

**Figure 4:**
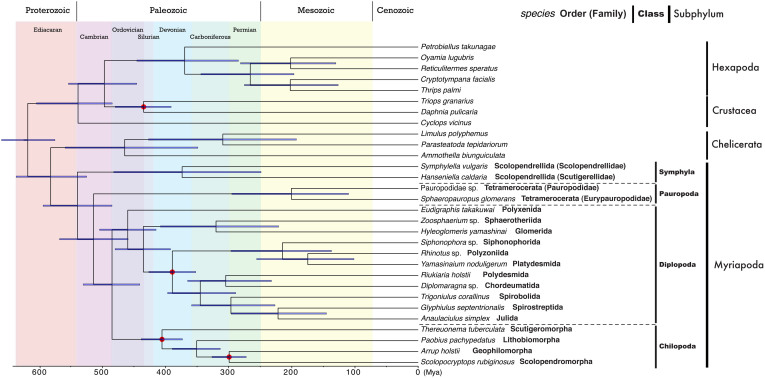
Estimated divergence time of myriapod clades. Blue bars across nodes indicate 95% highest posterior density of the node estimate for divergence time (refer to Supplementary Fig. S2). Red circles at nodes indicate fossil calibration points.

Divergence time was estimated using the BEAST^33^ with Bayesian inference based on data for four fossils. The tree topology was the same as the ML tree shown in Fig. 3, except for the relationships between the three colobognath diplopods (Fig. 4). The 95% highest posterior density (HPD) interval data for all nodes are shown in Supplementary Fig. S3. Divergence among the four myriapod classes was estimated to have occurred during the period from the early Cambrian to the early Ordovician. Divergence of the chilopod orders appears to have occurred some time between the Devonian and the early Permian (Fig. 4). Diversification of the Diplopoda dates back to the Ordovician, whereas the most closely related orders analyzed in this study separated in the middle of the Mesozoic. Interestingly, divergence between two families of Symphyla predates the divergence between orders of diplopods and chilopods (Fig. 4).

### Evolutionary patterns of post-embryonic development mode

**Figure 5:**
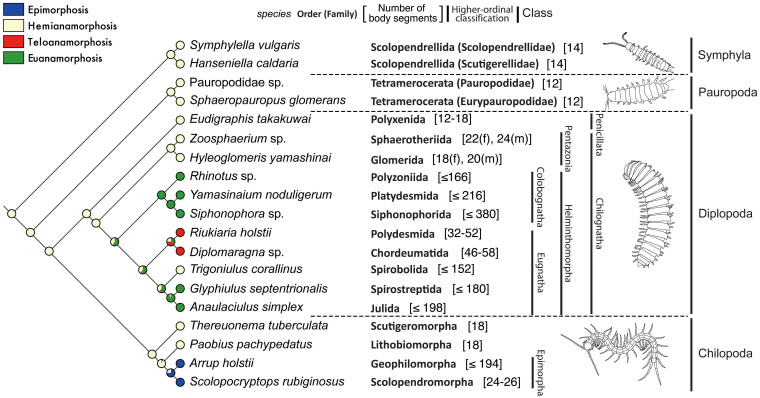
Evolutionary transition of post-embryonic development mode. Ancestral state estimation is based on the RAxML topology (Fig. 3) using a likelihood algorithm. Pie charts represent the relative likelihood of different mode of post-embryonic development. The number of body segments of the myriapods belonging to the order is shown in brackets after the order name. The illustrations of the four representative myriapods were drawn by the first author (H.M.) based on the pictures shown in Fig. 1.

Among extant myriapods, post-embryonic development is considered to occur by one of four modes: epimorphosis, euanamorphosis, hemianamorphosis, and teloanamorphosis^4^,^5^. Myriapods employing epimorphic and teloanamorphic modes were clustered into common groups, respectively, whereas myriapods employing the euanamorphic and hemianamorphic modes were separated into different clades (Fig. 5). Ancestral state estimation using Mesquite (a modular system for evolutionary analysis, Version 2.75. [http://mesquiteproject.org]) based on the inferred phylogenetic tree topologies revealed that hemianamorphic mode is the ancestral condition of myriapod post-embryonic development, and that the other modes developed from the hemianamorphic mode (Fig. 5). However, it is possible that the origin of the teloanamorphic mode may be the euanamorphic mode (Fig. 5). The likelihood proportions of the four modes on the nodes of the tree topology are shown in Supplementary Fig. S4.

Myriapods (excepting Spirobolida) with a hemianamorphic mode of post-embryonic development have markedly fewer body segments than taxa that follow other modes (Fig. 5). Thus, our results suggest that the ancestor of myriapods had few body segments and legs.

## Discussion

### Interclass relationships of myriapods

Among the four classes of myriapods, Symphyla, Diplopoda, and Pauropoda are considered to form a monophyletic group (Progoneata) based on shared morphological characters, including the anterior position of the gonopore, the fused labrum, the swollen trichobothria with a basal bulb, and other features^17^. Diplopoda and Pauropoda are further grouped into a taxon, Dignatha, based on morphological characters, including a limbless second maxillary segment, the position of spiracles^17^,^18^; of the interclass relationships that have been proposed to date, Dignatha is considered to be the least controversial taxon^34^.

However, phylogenies based on molecular data have been highly controversial^10^,^14^,^15^,^16^,^21^,^35^. For example, while three studies by Regier et al.^14^,^16^,^35^ strongly support the monophyly of each class, the interclass relationships proposed in these studies are inconsistent and all are weakly supported. Analysis using the ribosomal RNA gene suggests that Diplopoda is the sister lineage to other myriapods, and Symphyla and Pauropoda are sister taxa^15^. However, these authors also indicated that the grouping of pauropods and symphylids may have been due to LBA artifacts, as the two taxa were connected by very long branches in the tree. Phylogenomic and mitochondrial studies^10^,^21^ support the assignment of the taxon Progoneata and the sister relationship between Symphyla and Pauropoda. However, the relatively few samples and outgroups employed in the mitochondrial study^21^, combined with the very long branch lengths for the pauropod species, imply that the results need to be interpreted with caution. Some studies have proposed that it is difficult to resolve the relationships among the basal arthropod lineages using mitogenomic data alone, because the relationships inferred by these data are highly influenced by the choice of the outgroup, data treatment method, and the genes examined^36^,^37^,^38^. In the phylogenomic study^10^ of arthropods, the sister relationship of Symphyla to Pauropoda was strongly supported in nucleotide sequence-based analyses, while amino acid sequence-based analyses gave weak support for the relationship. However, some controversy exists regarding the reliability of results obtained from analyses based on nucleotide sequences^39^,^40^. Compared to previous studies, the present study proposes that Symphyla is the sister lineage to all other myriapod clades; analyses based on amino acid sequences gave strong support (Fig. 3) for this relationship and significantly rejected the previously proposed hypotheses (Table 1).

Recently, phylogenomics, the inference of phylogenetic relationships using genome-scale data, has increasingly become a powerful tool to resolve difficult phylogenetic question. However, the increase of non-phylogenetic signals in a large genomic dataset would give the misleading effect on phylogenetic analysis^41^. Indeed, three recent large-scale analyses for the early diversification of animals have shown discrepant results^41^. In the arthropod phylogenetic analyses using 62 nuclear protein-coding genes, the relationships among myriapod classes could not be resolved clearly due to the relatively low supports^10^. These results mean that using simply large multigene datasets does not necessarily resolve difficult phylogenetic issues. What is the most important in phylogenomic analysis would be how to reduce the non-phylogenetic signals from the large multigene datasets, in the other words, how to select the genes that are appropriate for phylogenetic analysis^41^. The three genes (DPD1, RPB1, and RPB2) used in the present analyses have effectively resolved the phylogenetic relationships of the higher groups of hexapods^30^,^31^, and it has been suggested that these genes could be considered useful for phylogenetic analyses of other arthropod groups. Our present results further confirmed the usefulness of these genes in phylogenetic analyses of arthropods, because the tree topology (Fig. 3) gave totally reasonable relationships of myriapods and strong support for most nodes of the tree topology. The series of the arthropod phylogenetic studies using the three nuclear protein-coding genes suggest that a few genes could also resolve some difficult phylogenetic questions.

Based on mouthpart structure, Sharov^42^ considered Symphyla and Hexapoda to be closely related, and named the taxon Dimalata, while the other myriapod classes, Chilopoda, Diplopoda, and Pauropoda were grouped into the taxon Monomalata. The morphological evidence for these groupings is as follows. Hexapoda and the Symphyla use the mandibles and the first maxillae for manipulation of the food while the second maxillae form the labium, whereas Chilopoda, Diplopoda and Pauropoda all have a pair of mandibles with masticatory function, while the maxillae (one or two pairs) form the posterior wall of the pre-oral cavity. However, such a close relationship between Hexapoda and Myriapoda has been rejected by molecular evidence, and Pancrustacea (Crustacea + Hexapoda) has become the consensus view. Nonetheless, the structure of the mouthparts of Symphyla does indeed differ from that of other myriapods, and this difference is consistent with the results of the current study.

The presence of pectinate lamellae on the distal part of the mandibular gnathal edge in members of Diplopoda and Chilopoda has been shown, and a common origin for the lamellae has been suggested, based on structural similarities such as the position, structure and orientation of these structures^43^,^44^. While the presence of lamellae in Pauropoda has not yet been clarified, the absence of these structures in the Symphyla has been confirmed^43^,^44^. These morphological characters support the present results, which proposes that Symphyla is a sister lineage to other myriapods.

### Chilopoda phylogeny

The class Chilopoda consists of five orders, Scutigeromorpha, Craterostigmomorpha, Lithobiomorpha, Geophilomorpha, and Scolopendromorpha. Unfortunately, Craterostigmomorpha was not included in the present analysis because we were unable to obtain fresh specimens and determine the target gene sequences using our genomic DNA. Both morphological^17^,^22^,^24^,^45^,^46^ and molecular phylogenetic analyses^10^,^16^,^26^,^45^,^46^ have suggested that Scutigeromorpha is the sister lineage to all other chilopod groups, and our results corroborate this relationship. In addition, morphological studies have also suggested that the orders Scolopendromorpha and Geophilomorpha are closely related, and have grouped these into a taxon named Epimorpha^17^. Among the molecular studies that have been conducted on these groups to date, some analyses support the validity of Epimorpha^17^,^45^,^46^, while others are more supportive of a sister relationship between Scolopendromorpha and Lithobiomorpha^17^,^26^, although no strong support values for the topologies were obtained from these analyses. The latter relationship would suggest that Scolopendromorpha and Geophilomorpha, which follow an epimorphic mode of post-embryonic development, evolved independently of the anamorphic chilopod lineages. Our data strongly support the validity of the taxon Epimorpha, i.e., a sister relationship between the two epimorphic orders Scolopendromorpha and Geophilomorpha, which suggests that the epimorphic chilopods have a common origin (Figs 3, 5). Our findings on the interordinal relationships of Chilopoda are congruent with the traditional view inferred from morphological characters^22^. Taking present results plus that previously inferred from a phylogenomic analysis of arthropods^10^ into consideration, the reasonable interordinal relationships of chilopods would be (Scutigeromorpha, (Craterostigmomorpha, (Lithobiomorpha, (Geophilomorpha, Scolopendromorpha)))).

### Diplopoda phylogeny

The subclass Chilognatha is classified into two infraclasses, Pentazonia and Helminthomorpha. Pentazonia is composed of three orders: Glomerida, Sphaerotheriida, and Glomeridesmida. Molecular analyses based on three nuclear genes inferred the relationships between these taxa to be (Sphaerotheriida, (Glomerida, Glomeridesmida)), although support for monophyly of Pentazonia was weak^14^. Our analyses provided strong support for a sister relationship between Glomerida and Sphaerotheriida, although Glomeridesmida was excluded from the analyses (Fig. 3). Taken together, these results imply that Pentazonia is a monophyletic group. The infraclass Helminthomorpha has been further divided into two subterclasses, Colobognatha and Eugnatha. Molecular evidence obtained by Regier et al. (2005) and this study supports the monophyly of Colobognatha; however, the internal phylogenetic relationships remain unclear (Fig. 3). The subterclass Eugnatha consists of three superorders, which are Nematophora (three orders: Chordeumatida, Callipodida, and Stemmiulida), Merocheta (one order: Polydesmida), and Juliformia (three orders: Spirobolida, Spirostreptida, and Julida)^4^. However, attempts to clarify the relationships among these superorders using morphological and molecular methods have produced ambiguous results^14^,^28^. Similarly, the results of the present study were also unable to resolve these relationships. However, the sister relationship between the two orders Spirostreptida and Julida was strongly supported by both ML and BI analyses in this study. Our analyses also support monophyly of the subterclass Eugnatha and the superorder Juliformia, although the bootstrap values were relatively low (Fig. 3). In addition, our results also strongly support the monophyly of two higher-level taxa, the subclass Chilognatha and the infraclass Helminthomorpha (Fig. 3).

### Divergence time

Analyses of myriapod fossils have placed the time of divergence between Chilopoda and Diplopoda at, or before, the mid-Silurian^47^. Molecular clock analysis has suggested that the time of divergence may go back to the late Cambrian^48^. The present analysis showed that the first myriapod split occurred between the class Symphyla and other classes, and not between Chilopoda and other classes. It is therefore not difficult to imagine that diversification of myriapods may in fact predate current estimates. In fact, our results support this assumption, and show that the divergence time between Symphyla and other myriapods dates back to the early Cambrian (Fig. 4). This would imply a higher number of water-to-land transitions than ever accepted thus far for arthropods, because in the Cambrian there were no soils, no terrestrial life, and the earliest fossil evidence for land plants are from the the Middle Ordovician^49^. In turn, this would likely imply parallel independent evolution of a diversity of specific adaptations of myriapods to the terrestrial environment. Symphyla consists of only one order containing two families, implying that Symphyla has low morphological diversity. Interestingly, the divergence time between the two families goes back to at least the Paleozoic (Fig. 4), which predates diversification among most of the myriapod orders. These findings suggest that the morphology of symphylids has remained relatively unchanged for more than 250 million years.

### Ancestral condition and evolutionary patterns of post-embryonic developoment mode in myriapods

The term *myriapod* means “myriad legs”. However, our present results show that having a “myriad legs” is not the ancestral condition of myriapods. Within the context of post-embryonic development, extant myriapods are characterized by two very important characteristics: the ability to molt and the ability to add segments, although the latter requires a molt. Except for two chilopod orders, Geophilomorpha and Scolopendromorpha, in which the formation of segments and legs is completed during embryonic development, all myriapods acquire more segments and legs during their post-embryonic development^5^. Four modes of post-embryonic development (hemianamorphosis, euanamorphosis, teloanamorphosis, and epimorphosis) are recognized in extant myriapods (see Fig. S1). In the tree topology inferred in this study, hemianamorphic myriapods were placed in basal clades (Fig. 5), and ancestral state estimation clearly demonstrated that the hemianamorphic mode is the ancestral condition and that the other modes are derived (Fig. 5). The hemianamorphic mode is also found in many members of Crustacea, Protura (a basal clade of Hexapoda), Pycnogonida (Chelicerata), some fossil chelicerates, and Trilobita^6^,^50^. These findings have suggested that hemianamorphic mode may represent the ancestral condition of post-embryonic development in Euarthropoda^5^,^6^,^50^. Therefore, present results support this view. Our results also showed that taxa with an euanamorphic mode have evolved from those with a hemianamorphic mode after diversification of the infraclass Helminthomorpha (class Diplopoda), and that taxa with a teloanamorphic mode have diverged later, probably from taxa following either euanamorphic or hemianamorphic modes (Fig. 5). In addition to our results, the order Spirostreptida contains euanamorphic species (suborders Cambalidea and Epinannolenidea) as well as hemianamorphic species (suborder Spirostreptidea)^4^,^5^. Therefore, the post-embryonic development mode in the taxon Helminthomorpha would have changed several times. Based on the likelihood proportions of the post-embryonic development modes on the nodes of the tree topology (Fig. 5 and Supplementary Fig. S4), two likely scenarios would be considered: (1) euanamorphosis has evolved independently and repeatedly from the hemianamorphic condition, and (2) euanamorphosis evolved from the hemianamorphic mode only one time at the ancestral stage of the Helminthomorpha, then teloanamorphosis derived from it and some helminthomorph clades returned to hemianamorphic mode. In addition, our results also clearly show that epimorphic chilopods (Scolopendromorpha and Geophilomorpha) are derived from the hemianamorphic chilopod lineage (Fig. 5).

In the tree topology inferred in this study, Helminthomorpha and Epimorpha form monophyletic clades within the classes Diplopoda and Chilopoda, respectively (Fig. 3). Compared to the myriapods of the more basal lineages, the myriapods within these two taxa have many more body segments (Fig. 5). In addition, taxa with a hemianamorphic mode (ancestral condition) of post-embryonic development have markedly fewer body segments than taxa that follow other modes (Fig. 5). These results suggest that the ancestor of myriapods had a small number of segments and legs. In other words, an increase in segment numbers has occurred in the evolutionary process in myriapods. Fusco^5^ has mentioned that evolutionary change toward considerably higher numbers of segments has occurred at least twice: in helminthomorph millipedes and in the epimorphic centipede clade Geophilomorpha. The present results completely support this view.

## Methods

### Samples

In total, 19 myriapod species representing all four classes (11 diplopod orders, 4 chilopod orders, 2 pauropod orders and 2 symphylid families) were used in this study (Table 2). These samples were mainly collected on the Ryukyu Islands and in the Kansai region of Japan, and were maintained alive for RNA extraction. One species, *Glyphiulus septentrionalis* was stored in *RNAlater* solution (Ambion) for several days until RNA extraction. As outgroups for phylogenetic analysis, 3 chelicerate species were sequenced in this study, and the sequences of 3 crustaceans and 5 hexapods sequenced in our previous studies^30^,^31^ were also used (Table 2).

### RNA extraction, RT-PCR and sequencing

Total RNA was extracted from living samples, and the specimen stored in *RNAlater* solution (Ambion) using an ISOGEN kit (Nippon Gene). The total RNA was reverse-transcribed to cDNA using a SMART RACE cDNA Amplification Kit (Clontech) and SuperScript III Reverse Transcriptase (Invitrogen). The cDNA samples were then used as templates for PCR amplification with TaKaRa LA-Taq (Takara Bio Inc.) using sense and antisense degenerate primers for the three target genes, DPD1, RPB1, and RPB2, as described previously^30^. The amplification conditions were 94°C for 3 min, followed by 30 cycles of 95°C for 30 sec, 50°C for 30 sec, 72°C for 4 min, and extension at 72°C for 8 min. The PCR products were purified and directly sequenced using an ABI 3130xl Genetic Analyzer (Applied Biosystems).

### Sequence alignment and phylogenetic analyses

The sequence data of the 3 genes from the 19 myriapods and the 11 outgroup species were used in the phylogenetic analyses. The predicted amino acid sequences were aligned using MAFFT L-INS-i^51^ and ambiguous sites were removed using Gblocks^52^ with default parameters. The best-fit model for phylogenetic analyses was determined based on each sequence alignment using ProtTest 3 under the AIC, BIC, and AICc criteria^53^. Variable and parsimony-informative aa sites in the alignments were calculated with MEGA^54^. The sequence alignments of the 3 genes were finally concatenated to one sequence alignment for phylogenetic analysis. Maximum likelihood (ML) analysis was carried out with RAxML v7.2.8^55^ under the following models (using *-q* option): LG model for RPB1 and RPB2, and WAG model for DPD1, and the topology was assessed by performing 1,000 nonparametric bootstrap replicates. Bayesian inference (BI) was performed with MrBayes v3.2 under the WAG + gamma model and run for 50,000,000 generations^56^. The log file of the MrBayes analyses was examined by calculating the effective sample sizes (ESS) of all parameters using Tracer v1.5 (http://tree.bio.ed.ac.uk/software/tracer/). The sequence alignment used in the phylogenetic analyses and the ML tree topology have been deposited in TreeBase with accession URL (http://purl.org/phylo/treebase/phylows/study/TB2:S14525).

The approximately unbiased (AU), Kishino–Hasegawa (KH), Shimodaira-Hasegawa (SH), weighted Kishino-Hasegawa (wKH), and weighted Shimodaira–Hasegawa (wSH) tests were conducted using CONSEL^32^ to compare the topologies based on the alternative hypotheses that have been proposed in previous studies.

### Estimation of divergence time and ancestral state

Divergence time was estimated using the BEAST v1.7.5 program^33^, and the configurations were as follows: uncorrelated relaxed clock model, birth-death process for tree prior, random starting tree. Four fossil calibration points were set using a normal prior distribution; the fossil ages were set to the means, and the standard deviation (SD) was set to give confidence ±5% of fossil age. The bifurcation of *Triops granarius* and *Daphnia pulicaria* was set to 490 Mya (mean) and 24.5 (SD), from the unambiguous fossils of branchiopods in the Late Cambrian^57^. The basal split of Chilopoda was set to 418 Mya (mean) and 20.9 (SD), from the Scutigeromorpha fossils in the Late Silurian^58^. The split of Scolopendromorpha and Geophilomorpha was set to 306 Mya (mean) and 15.3 (SD), from the Scolopendromorpha fossils in the Upper Carboniferous^59^. The diversification of helminthomorphs was set to 430 Mya (mean) and 21.5 (SD), from the fossil of *Cowiedesmus eroticopodus* in the mid Silurian^47^. The root height, indicating diversification of Arthropoda, was set to 520 Mya (mean) and 26 (SD)^58^. The analysis was run for 20,000,000 generations with sampling at every 1,000 generations. The log file of the BEAST analysis was examined by calculating the effective sample sizes (ESS) of all parameters using Tracer v1.5 (http://tree.bio.ed.ac.uk/software/tracer/).

The ancestral condition of the post-embryonic development mode^5^,^6^ in myriapods was estimated with Mesquite v2.75 (http://mesquiteproject.org) using the Mk1 (Markov k-state 1 parameter) model.

## Author Contributions

H.M. and Z.-H.S. designed the study, collected samples, contributed to the discussion and wrote the paper. H.M. sequenced genes from myriapods and performed data analyses. C.U. sequenced genes from chelicerates. K.Y. contributed to sampling, identification and discussions.

## Supplementary Material

Supplementary Information suppl_info

## Figures and Tables

**Figure 1:**
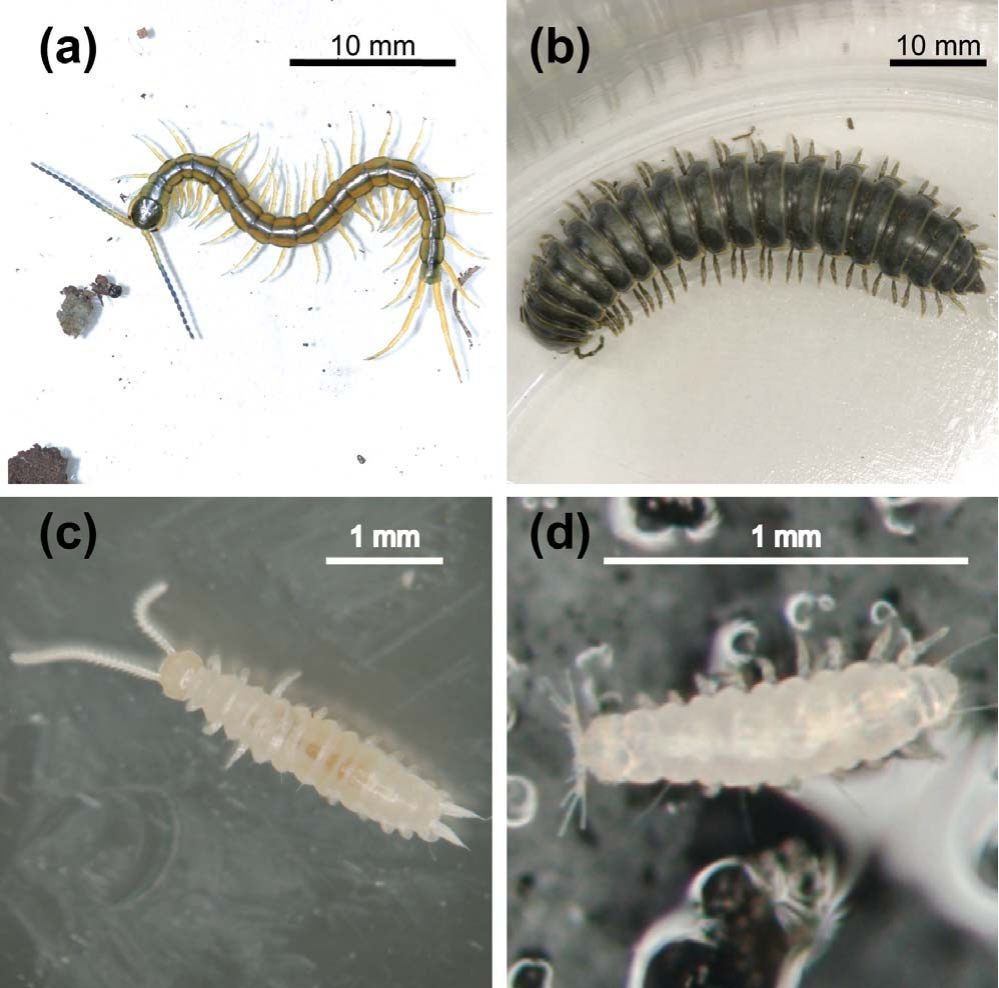
Representatives of four myriapod classes. (a) *Scolopendra* sp. (Chilopoda). (b) *Riukiaria* *holstii* (Diplopoda). (c) *Hanseniella caldaria* (Symphyla). (d) Pauropodidae sp. (Pauropoda). These pictures were taken by the first author (H.M.).

**Table 1:** Statistical testing of the hypotheses on the interclass relationships of Myriapoda

						p-value		
Tree		Topology	-In L	AU	KH	SH	wKH	wSH
Fig. 3	Our tree topology	(Symphyla, (Pauropoda, (Chilopoda, Diplopoda)))	36539.21707	0.999	0.995	0.996	0.992	0.995
Fig. 2a	Progoneata-Dignatha hypothesis	(Chilopoda, (Symphyla, (Diplopoda, Pauropoda)))	36578.67945	<0.001	0.002	0.002	0.008	0.008
Fig. 2b	Trignatha-Dignatha hypothesis	((Chilopoda, Symphyla), (Pauropoda, Diplopoda))	36578.67282	0.004	0.010	0.010	0.010	0.018
Fig. 2c	Progoneata-Edafopoda hypothesis	(Chilopoda, (Diplopoda, (Pauropoda, Symphyla)))	36585.36848	0.001	0.005	0.005	0.005	0.008

AU, the approximately unbiased test calculated from the multiscale bootstrap; KH, the Kishino-Hasegawa test; SH, the Shimodaira-Hasegawa test; wKH, the weighted Kishino-Hasegawa test; wSH, the weighted Shimodaira-Hasegawa test.

**Table 2:** A list of taxa used for phylogenetic analyses in this study

						DDBJ/EMBL/GenBank accession number		
Subphylum	Class	Order	Species	Mode*	Locality	DPD1	RPB1	RPB2
Myriapoda	Chilopoda	Scutigeromorpha	*Thereuonema tuberculata*	Hemi	Yodo River, Settsu, Osaka, Japan	AB831769	AB831770	AB831771
		Lithobiomorpha	*Paobius pachypedatus*	Hemi	Tamagusuku, Nanjo, Okinawa, Japan	AB831742	AB831743	AB831744
		Scolopendromorpha	*Scolopocryptops rubiginosus*	Epi	Nariaiminaminomachi, Takatsuki, Osaka, Japan	AB831757	AB831758	AB831759
		Geophilomorpha	*Arrup holstii*	Epi	Chinen, Nanjo, Okinawa, Japan	AB831748	AB831749	AB831750
	Diplopoda	Polyxenida	*Eudigraphis takakuwai*	Hemi	Tamagusuku, Nanjo, Okinawa, Japan	AB831727	AB831728	AB831729
		Sphaerotheriida	*Zoosphaerium* sp.	Hemi	Madagascar	AB831778	AB831779	AB831780
		Glomerida	*Hyleoglomeris yamashinai*	Hemi	Tamagusuku, Nanjo, Okinawa, Japan	AB831736	AB831737	AB831738
		Polyzoniida	*Rhinotus* sp.	Eu	Mt. Banna, Ishigaki, Okinawa, Japan	AB831751	AB831752	AB831753
		Platydesmida	*Yamasinaium noduligerum*	Eu	Ooharanooshio, Saikyo-ku, Kyoto, Japan	AB831775	AB831776	AB831777
		Siphonophorida	*Siphonophora* sp.	Eu	Isen-cho, Tokunoshima, Kagoshima, Japan	AB831760	AB831761	AB831762
		Chordeumatida	*Diplomaragna* sp.	Telo	Nariaiminaminomachi, Takatsuki, Osaka, Japan	AB831724	AB831725	AB831726
		Polydesmida	*Riukiaria holstii*	Telo	Mt. Nago, Nago, Okinawa, Japan	AB831754	AB831755	AB831756
		Spirostreptida	*Glyphiulus septentrionalis*	Eu	Yara, Kadena, Okinawa, Japan	AB831730	AB831731	AB831732
		Julida	*Anaulaciulus simplex*	Eu	Mt. Yoza, Itoman, Okinawa, Japan	AB831721	AB831722	AB831723
		Spirobolida	*Trigoniulus corallinus*	Hemi	Ishikawasonan, Uruma, Okinawa	AB831772	AB831773	AB831774
	Pauropoda	Tetramerocerata	Pauropodidae sp.	Hemi	Yamate-cho, Takatsuki, Osaka, Japan	AB831745	AB831746	AB831747
			*Sphaeropauropus glomerans*	Hemi	Minase River, Shimamoto, Osaka, Japan	AB831766	AB831767	AB831768
	Symphyla	Scolopendrellida	*Symphylella vulgaris*	Hemi	Yamate-cho, Takatsuki, Osaka, Japan	AB831763	AB831764	AB831765
			*Hanseniella caldaria*	Hemi	Minase River, Shimamoto, Osaka, Japan	AB831733	AB831734	AB831735
Chelicerata	Arachnida	Araneae	*Parasteatoda tepidariorum*		Murasaki-cho, Takatsuki, Osaka, Japan	AB831715	AB831716	AB831717
	Merostomata	Xiphosura	*Limulus polyphemus*		America	AB831739	AB831740	AB831741
	Pycnogonida	Pantopoda	*Ammothella biunguiculata*		Shirahama-cho, Wakayama, Japan	AB831718	AB831719	AB831720
Crustacea	Maxillopoda	Cyclopoida	*Cyclops vicinus*		Kawashima-cho, Aichi, Japan	AB811980	AB811994	BAJ78715
	Branchiopoda	Cladocera	*Daphnia pulicaria*		Takiwaki-cho, Toyota, Aichi, Japan	AB811983	AB811997	AB812011
	Branchiopoda	Notostraca	*Triops granarius*		Ehime, Japan	AB811984	AB811998	AB812012
Hexapoda	Insecta	Hemiptera	*Cryptotympana facialis*		Murasaki-cho, Takatsuki, Osaka, Japan	AB598719	AB596918	AB597609
	Insecta	Plecoptera	*Oyamia lugubris*		Higashigawa-machi, Matsuyama, Ehime, Japan	AB598710	AB596909	AB597600
	Insecta	Archaeognatha	*Petrobiellus takunagae*		Shiroyama, Shimoda, Shizuoka, Japan	AB598695	AB596894	AB597585
	Insecta	Isoptera	*Reticulitermes speratus*		Tarumi, Matsuyama, Ehime, Japan	AB598716	AB596915	AB597606
	Insecta	Thysanoptera	*Thrips palmi*		Shimoidai-machi, Matsuyama, Ehime, Japan	AB598717	AB596916	AB597607

*, Mode of post-embryonic development. Epi, epimorphosis; Eu, euanamorphosis; Hemi, hemianamorphosis; Telo, teloanamorphosis.
