# Supplementary material for: Molecular phylogeny of Myriapoda provides insights into evolutionary patterns of the mode in post-embryonic development
Source: Sci Rep. 2014 Feb 18;4:4127. doi: 10.1038/srep04127 (PMC3927213; doi:10.1038/srep04127)
Supplement: Supplementary Information — suppl_info [file srep04127-s1.pdf]

Molecular phylogeny of Myriapoda provides insights into evolutionary patterns of the mode in post-embryonic development

Hideyuki Miyazawa[1,2], Chiaki Ueda[1,2], Kensuke Yahata[3] & Zhi-Hui Su[1,2,*]

[1] Department of Biological Sciences, Graduate School of Science, Osaka University, Osaka 560-0043, Japan. [2] JT Biohistory Research Hall, 1-1 Murasaki-cho, Takatsuki, Osaka 569-1125, Japan. [3] Graduate School of Life and Environmental Sciences, University of Tsukuba, Tsukuba, Ibaraki 305-8572, Japan.

*Correspondence and requests for materials should be addressed to Z.-H. S. (email: su.zhihui@brh.co.jp).

Supplementary Information

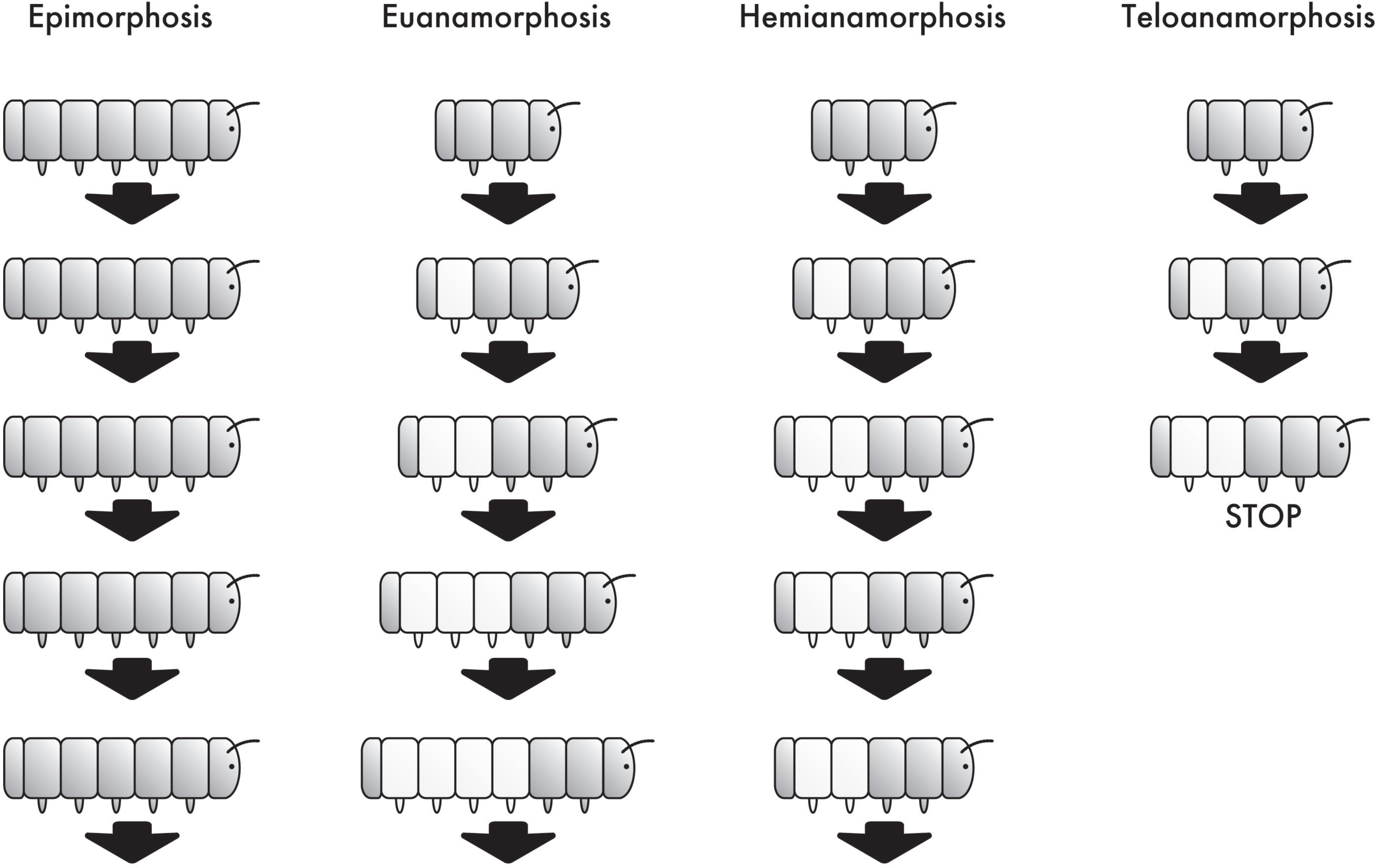

Supplementary Fig. S1. Four modes of metamorphosis during post-embryonic development in myriapods. For details, refer to the text. Modified by the first author (H.M.) based on Minelii and Fusco (2013).
Minelli, A. & Fusco, G. in Arthropod Biology and Evolution: Molecules, Development, Morphology (Springer, 2013).

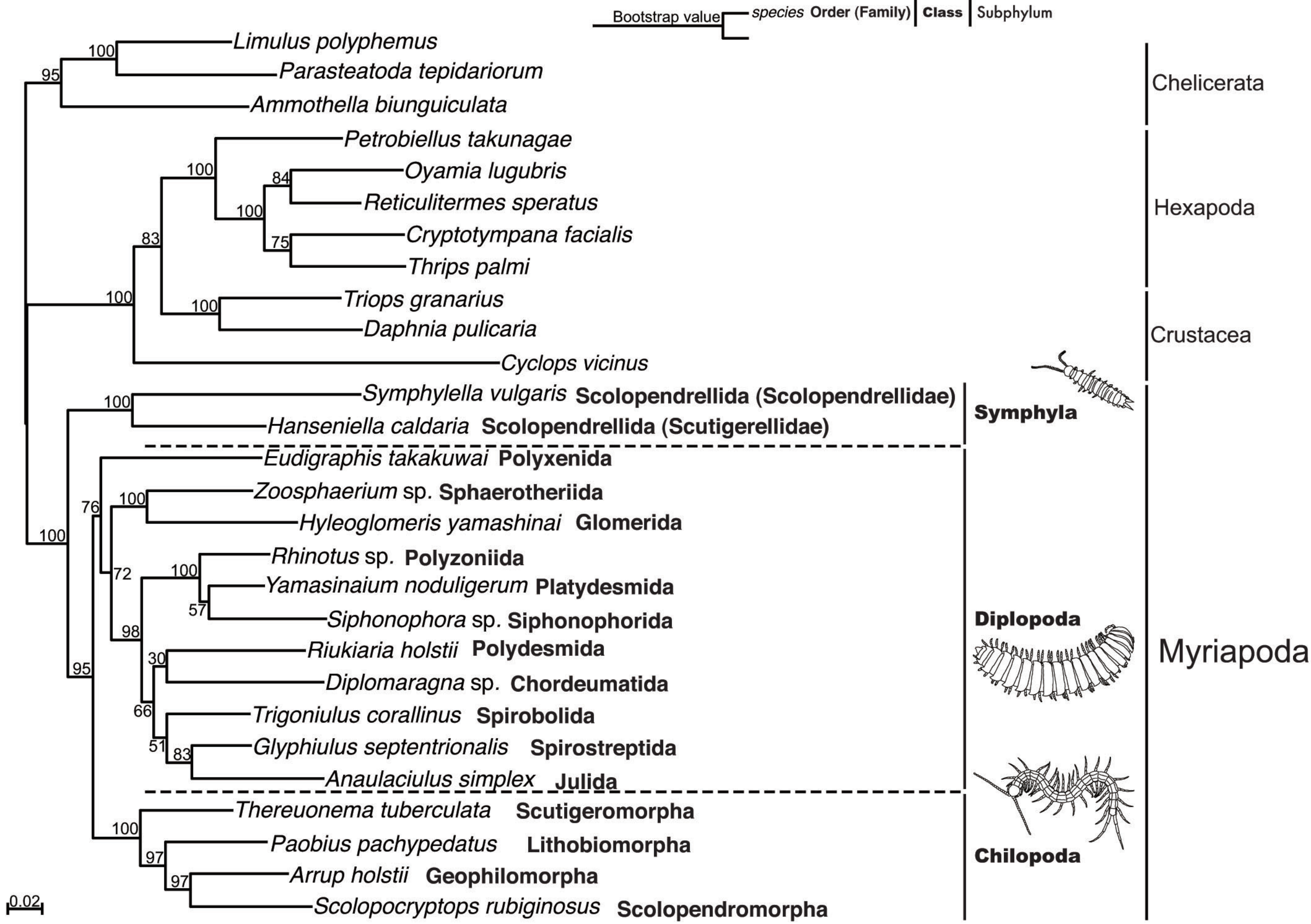

Supplementary Fig. S2. ML tree of myriapods excluding pauropods based on the combined amino acid sequences of DPD1, RPB1, and RPB2. Bootstrap values are shown at each node. Bold letters after the species name indicate order names. The illustrations of the three representative myriapods were drawn by the first author (H.M.) based on the pictures shown in Fig. 1.

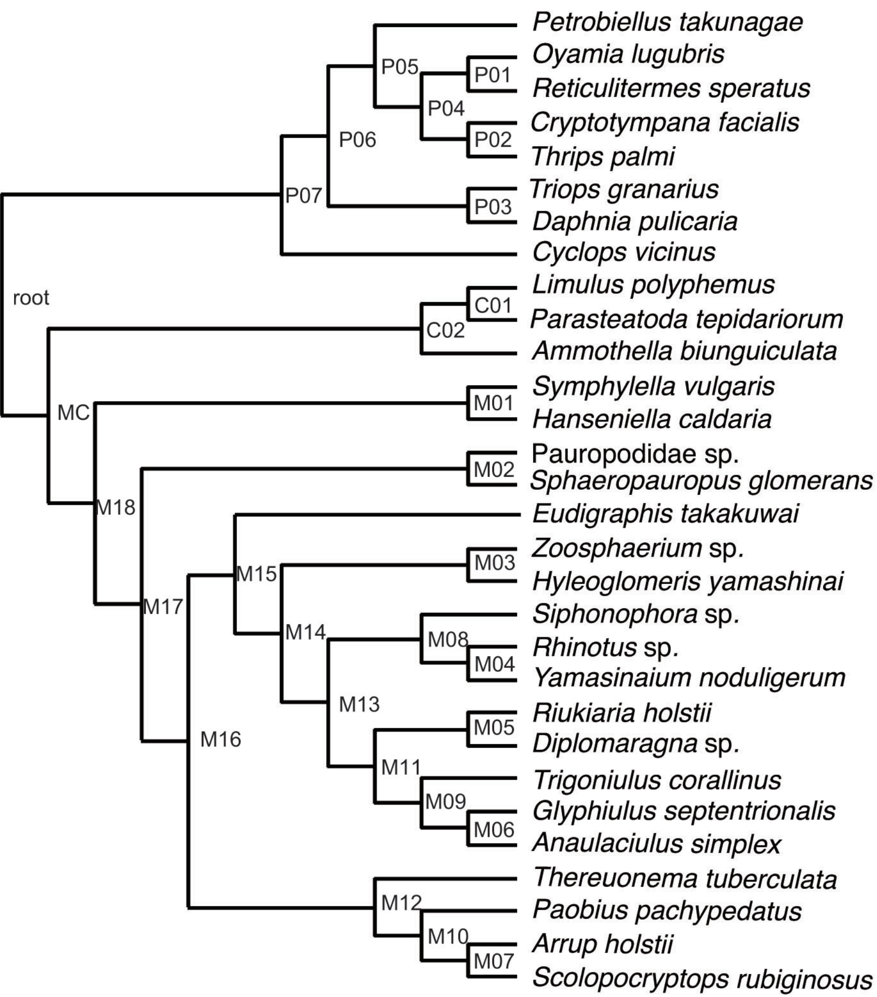

Number	Topology	Median	95% HPD	
M01	(24,10)	373.35	248.44	481.54
M02	(15,23)	200.397	109.88	294.61
M03	(30,11)	319.682	220.56	407.72
M04	(19,29)	174.383	101.29	255.27
M05	(7,20)	304.269	231.93	364.54
M06	(3,9)	221.508	144.78	295.78
M07	(17,21)	298.905	271.93	326.01
M08	((19,29),22)	214.591	136.85	296.04
M09	((3,9),27)	296.091	226.38	358.37
M10	(13,(17,21))	350.268	313	388.85
M11	((7,20),((3,9),27))	344.661	288.46	396.3
M12	((13,(17,21)),25)	405.215	372.81	438.06
M13	(((19,29),22),((7,20),((3,9),27)))	388.867	351.72	426.07
M14	((((19,29),22),((7,20),((3,9),27))),(30,11))	434.733	391.81	479.44
M15	(8,((((19,29),22),((7,20),((3,9),27))),(30,11)))	459.385	414.58	504.09
M16	(((13,(17,21)),25),(8,((((19,29),22),((7,20),((3,9),27))),(30,11))))	484.341	440.39	530.03
M17	((((13,(17,21)),25),(8,((((19,29),22),((7,20),((3,9),27))),(30,11)))),(15,23))	513.471	458.84	567.41
M18	((24,10),((((13,(17,21)),25),(8,((((19,29),22),((7,20),((3,9),27))),(30,11)))),(15,23)))	539.069	484.39	593.42
C01	(15,12)	308.872	192.37	426.55
C02	((15,12),2)	464.251	348.45	558.58
MC	(((10,24),(((((21,1),14),25),(8,(((((3,9),27),(7,20)),(22,(29,19))),(11,30)))),(16,23))),((15,12),2))	581.453	524.46	636.18
P01	(14,18)	201.489	129.99	280.95
P02	(4,26)	202.15	126.33	274.75
P03	(6,28)	434.263	390.8	479.21
P04	((13,18),(4,26))	265.521	196.33	343.58
P05	(16,((13,18),(4,26)))	369.261	284.21	444.95
P06	((6,28),(16,((13,18),(4,26))))	496.498	445.21	553.36
P07	(((6,28),(16,((13,18),(4,26)))),5)	538.114	484.03	603.98
root		617.652	574.76	659.15

1,*Parasteatoda tepidariorum*; 2,*Ammothella biunguiculata*; 3,*Anaulaciulus simplex*; 4,*Cryptotympana facialis*; 5,*Cyclops vicinus*; 6,*Daphnia pulicaria*; 7,*Diplomaragna* sp.; 8,*Eudigraphis takakuwai*; 9,*Glyphiulus septentrionalis*; 10,*Hanseniella caldaria*; 11,*Hyleoglomeris yamashinai*; 12,*Limulus polyphemus*; 13,*Paobius pachypedatus*; 14,*Oyamia lugubris*; 15,Pauropodidae sp.; 16,*Petrobiellus takunagae*; 17,*Arrup holstii*; 18,*Reticulitermes speratus*; 19,*Rhinotus* sp.; 20,*Riukiaria holstii*; 21,*Scolopocryptops rubiginosus*; 22,*Siphonophora* sp.; 23,*Sphaeropauropus glomerans*; 24,*Symphylella vulgaris*; 25,*Thereuonema tuberculata*; 26,*Thrips palmi*; 27,*Trigoniulus corallinus*; 28,*Triops granarius*; 29,*Yamasinaium noduligerum*; 30,*Zoosphaerium* sp.

Supplementary Fig. S3. Means and 95% highest posterior densities for all nodes from the divergence time estimation.

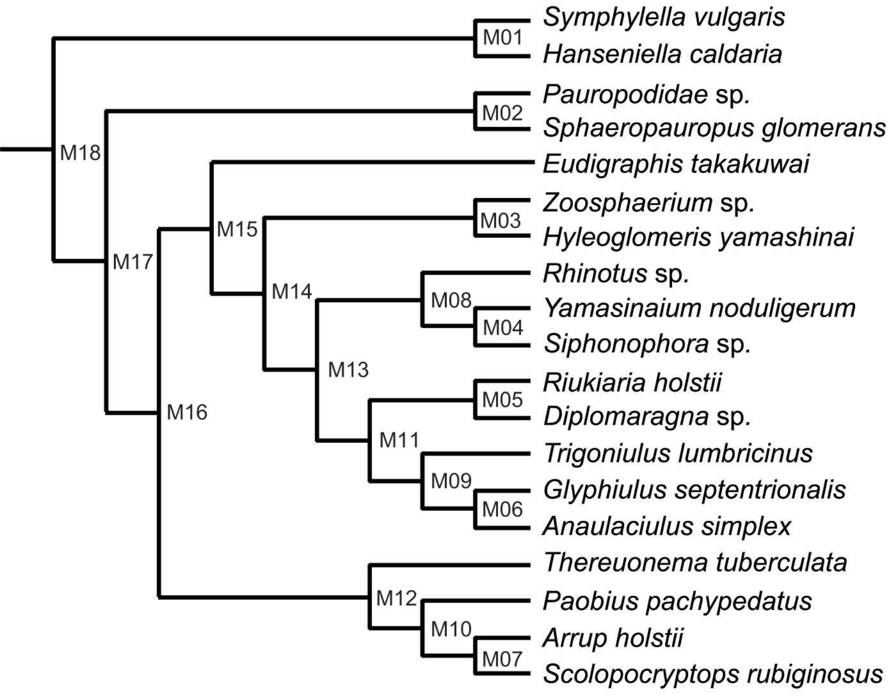

		Likelihood proportions			
Node No.	Topology	Epi	Hemi	Telo	Eu
M01	(6,15)	0.0011	0.9968	0.0011	0.0011
M02	(9,14)	0.0009	0.9972	0.0009	0.0009
M03	(7,19)	0.0004	0.9967	0.0005	0.0025
M04	(10,18)	0.0001	0.0019	0.0003	0.9977
M05	(11,18)	0.0058	0.2325	0.4547	0.3070
M06	(2,5)	0.0019	0.0813	0.0090	0.9078
M07	(1,12)	0.7333	0.2569	0.0049	0.0049
M08	((10,18),13)	0.0003	0.0050	0.0006	0.9941
M09	((2,5),17)	0.0015	0.4013	0.0370	0.5601
M10	((1,12),8)	0.0543	0.9430	0.0013	0.0013
M11	((11,18),((2,5),17))	0.0013	0.4039	0.0586	0.5362
M12	(((1,12),8),16)	0.0064	0.9930	0.0003	0.0003
M13	(((10,18),13),((11,18),((2,5),17)))	0.0014	0.4273	0.0355	0.5358
M14	((7,19),(((10,18),13),((11,18),((2,5),17))))	0.0002	0.9900	0.0008	0.0090
M15	(((7,19),(((10,18),13),((11,18),((2,5),17)))),4)	0.0001	0.9987	0.0001	0.0011
M16	((((7,19),(((10,18),13),((11,18),((2,5),17)))),4),(((1,12),8),16))	0.0001	0.9997	0.0000	0.0002
M17	((9,14),((((7,19),(((10,18),13),((11,18),((2,5),17)))),4),(((1,12),8),16)))	0.0002	0.9993	0.0002	0.0003
M18	((6,15),((9,14),((((7,19),(((10,18),13),((11,18),((2,5),17)))),4),(((1,12),8),16))))	0.0009	0.9973	0.0009	0.0009

Epi, epimorphosis; Hemi, hemianamorphosis; Eu, euanamorphosis; Telo, teloanamorphosis.

1, *Arrup holstii*; 2, *Anaulaciulus simplex*; 3, *Diplomaragna* sp.; 4, *Eudigraphis takakuwai*; 5, *Glyphiulus septentrionalis*; 6, *Hanseniella caldaria*; 7, *Hyleoglomeris yamashinai*; 8, *Paobius pachypedatus*; 9, Pauropodidae sp.; 10, *Rhinotus* sp.; 11, *Riukiaria holstii*; 12, *Scolopocryptops rubiginosus*; 13, *Siphonophora* sp.; 14, *Sphaeropauropus glomerans*; 15, *Symphylella vulgaris*; 16, *Thereuonema tuberculata*; 17, *Trigoniulus corallinus*; 18, *Yamasinaium noduligerum*; 19, *Zoosphaerium* sp.;

Supplementary Fig. S4. The likelihood proportions of the four modes of post-embryonic development on the nodes of the tree topology.
